# Supplementary material for: Room-temperature sub-100 nm Néel-type skyrmions in non-stoichiometric van der Waals ferromagnet Fe3-xGaTe2 with ultrafast laser writability
Source: Nat Commun. 2024 Feb 3;15:1017. doi: 10.1038/s41467-024-45310-2 (PMC10838308; doi:10.1038/s41467-024-45310-2)
Supplement: Supplementary file 1 — Supplementary Information [file 41467_2024_45310_MOESM1_ESM.pdf]

Supplementary Information for

Room-temperature sub-100 nm Néel-type skyrmions in non-stoichiometric van der Waals ferromagnet $Fe_{3-x}GaTe_2$ with ultrafast laser writability

Authors: Zefang Li[1#], Huai Zhang[2#], Guanqi Li[3#], Jiangteng Guo[1], Qingping Wang[4], Ying Deng[1], Yue Hu[1], Xuange Hu[1], Can Liu[1], Minghui Qin[2], Xi Shen[5], Richeng Yu[5], Xingsen Gao[2], Zhimin Liao[6], Junming Liu[2,7], Zhipeng Hou[2*], Yimei Zhu[8*] & Xuewen Fu[1,9*]

Affiliations:

[1] Ultrafast Electron Microscopy Laboratory, The MOE Key Laboratory of Weak-Light Nonlinear Photonics, School of Physics, Nankai University, Tianjin 300071, China

[2] Guangdong Provincial Key Laboratory of Optical Information Materials and Technology, Institute for Advanced Materials, South China Academy of Advanced Optoelectronics, South China Normal University, Guangzhou, 510006, China

[3] School of Integrated Circuits, Guangdong University of Technology, Guangzhou 510006, China

[4] College of Electronic information and automation, Aba Teachers University, Pixian Street, Wenchuan, 623002 China

[5] Beijing National Laboratory for Condensed Matter Physics, Institute of Physics, Chinese Academy of Sciences, Beijing, 100190 China

[6] State Key Laboratory for Mesoscopic Physics and Frontiers Science Center for Nano-optoelectronics, School of Physics, Peking University, Beijing 100871, China

[7] Laboratory of Solid State Microstructures and Innovation Center of Advanced Microstructures, Nanjing University, Nanjing 211102, China

[8] Condensed Matter Physics and Materials Science Department, Brookhaven National Laboratory, Upton, New York 11973, United States

[9] School of Materials Science and Engineering, Smart Sensing Interdisciplinary Science Center, Nankai University, Tianjin 300350, China

#These authors contribute equally to this work.

*Corresponding authors:
houzp@m.scnu.edu.cn (Z.H.); zhu@bnl.gov (Y.Z.); xwfu@nankai.edu.cn (X.F.).

1. Single crystal growth by Te-flux method.

Supplementary Note 1: We systematically grew a series of $Fe_{3-x}GaTe_2$ single crystals by varying the Fe content in the raw material composition, utilizing a Te-flux method. Subsequently, comprehensive energy-dispersive X-ray spectroscopy (EDS) mapping was conducted on the cleaved surfaces of these crystals to determine their chemical composition. To ensure the reliability of the EDS results, mapping was carried out at four distinct areas for each sample. Utilizing the Ga ratio as the normalization factor and ignoring the variation of Te content, their corresponding chemical composition were established. Table S1 provides a comprehensive overview of the raw material composition and the final crystal composition. It is clearly observed that when the raw Fe ratios fall below 0.8, the $Fe_{3-x}GaTe_2$ phase cannot be formed. Instead, a mixture of phases, including GaTe and Ga_2Te_3, is produced. When the raw Fe ratios are equal to or greater than 0.9, $Fe_{3-x}GaTe_2$ single crystals can be crystallized, with the Fe content in these single crystals increasing proportionally with the raw Fe ratios. However, an increase in the raw Fe ratios to 1.3 results in the formation of FeTe phases. In summary, we find that the chemical formulas for the crystals with the maximum and minimum Fe content correspond to $Fe_{2.84\pm0.05}GaTe_2$ and $Fe_{2.96\pm0.02}GaTe_2$, respectively. This result indicates that Fe vacancies always exist in the single crystals synthesized using a Te-flux method.

Table S1. Summary of the raw material composition and the final product for the growth of $Fe_{3-x}GaTe_2$ samples using the self-flux method.

Molar ratio of Fe: Ga: Te	Mass of Fe (g)	Mass of Ga (g)	Mass of Te (g)	Product
0.6:1:2	0.9349	1.9452	7.1200	GaTe, Ga_2Te_3
0.7:1:2	1.0739	1.9153	7.0107	
0.8:1:2	1.2088	1.8864	6.9048	
0.9:1:2	1.3397	1.8583	6.8020	$Fe_{2.84\pm0.05}GaTe_2$
1.0:1:2	1.4667	1.8310	6.7023	$Fe_{2.91\pm0.04}GaTe_2$
1.1:1:2	1.5900	1.8046	6.6054	$Fe_{2.95\pm0.03}GaTe_2$
1.2:1:2	1.7099	1.7789	6.5113	$Fe_{2.96\pm0.02}GaTe_2$
1.3:1:2	1.8263	1.7539	6.4198	FeTe

2. Detailed EDS mapping and HAADF image.

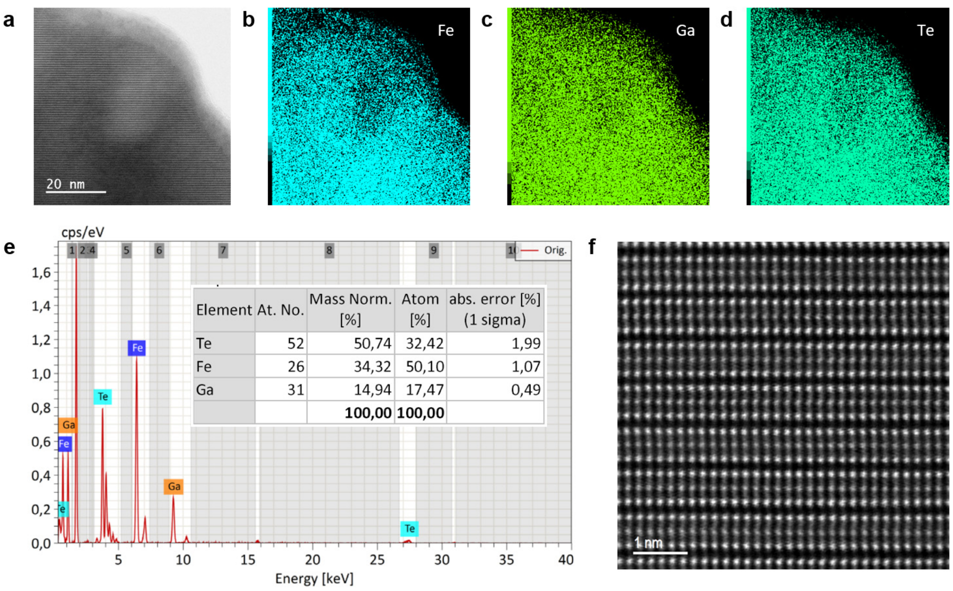

Fig. S1. | EDS and HAADF-STEM characterizations. a STEM images of a FIBed $Fe_{3-x}GaTe_2$ lamella along the [100] plane. **b-d** Corresponding EDS mapping of Fe, Ga, Te elements, respectively. **e** EDS spectrum and quantification results of $Fe_{2.84\pm0.05}GaTe_2$. **f** The HAADF image along [100] zone axis. The Fe-Ga-Te atom columns straightly lie along *c* axis, indicating no obvious lattice distortion in direction of *a* axis.

3. Detailed field-dependent magnetization.

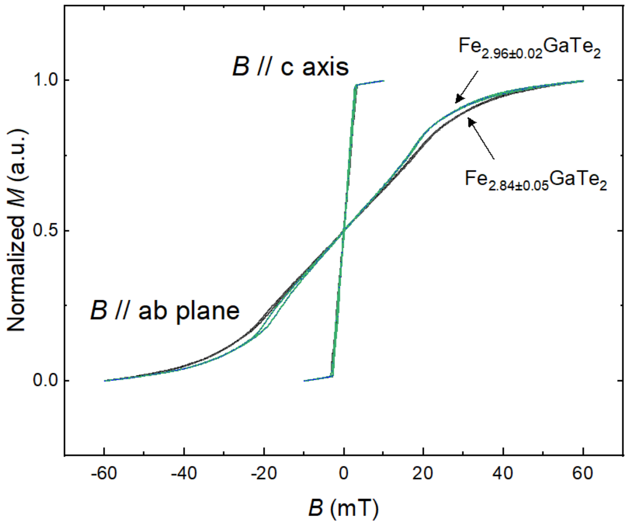

Fig. S2. | Field-dependent magnetizations. Room-temperature field-dependent magnetization curves for $Fe_{2.84\pm0.05}GaTe_2$ and $Fe_{2.96\pm0.02}GaTe_2$ samples.

4. Crystal structure of centrosymmetric Fe_3GaTe_2.

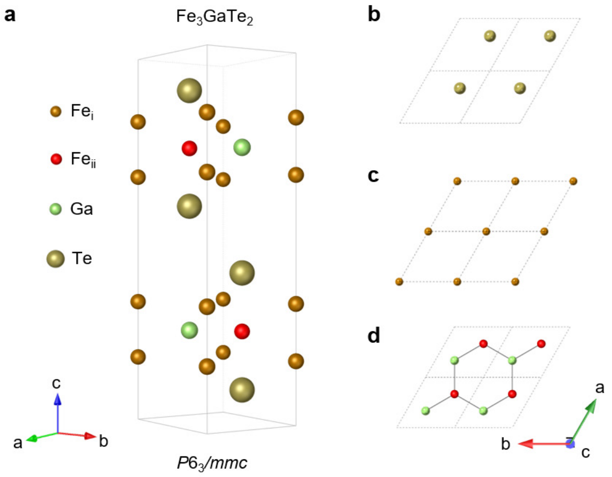

Fig. S3. | Schematic diagram of Fe_3GaTe_2 crystal structure. **a** Crystal structure of stoichiometric Fe_3GaTe_2. Each monolayer is sandwiched by Te-Fe_i-Fe_{ii}Ga-Fe_i-Te atom slices. **b** Top view of the Te atoms slice. **c** Top view of the Fe_i atoms slice. **d** Top view of the Fe_{ii}Ga atoms slice.

5. STEM image analysis of $Fe_{2.84\pm0.05}GaTe_2$.

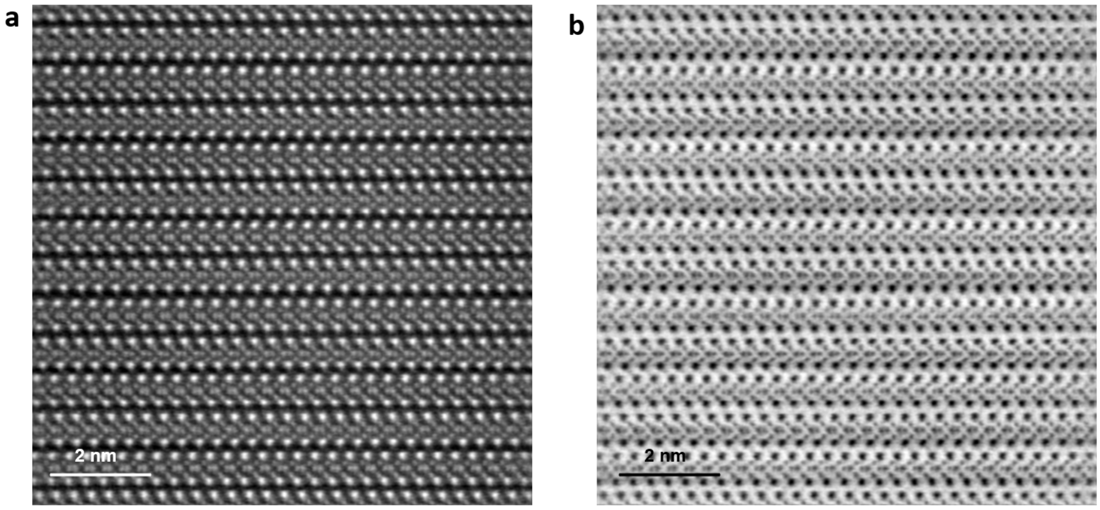

Fig. S4. | HR-STEM characterizations. **a** High-angle annular dark-field scanning transmission electron microscopy (HAADF-STEM) image and **b** annular bright-field scanning transmission electron microscopy (ABF-STEM) image of the $Fe_{2.84\pm0.05}GaTe_2$ sample along the $[11\bar{2}0]$ zone axis.

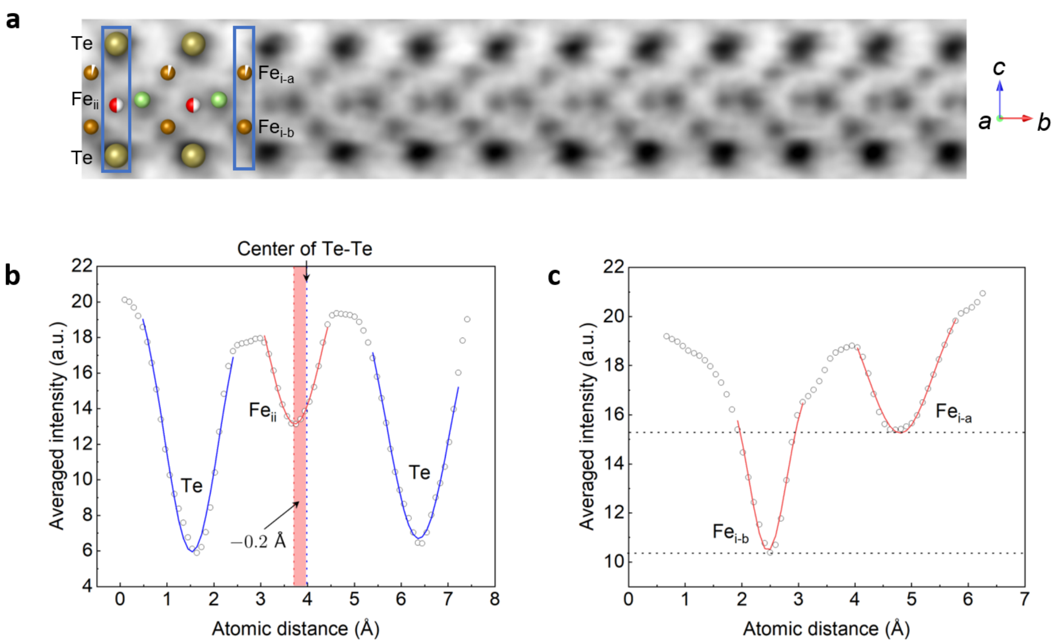

Fig. S5. | Detailed STEM image analysis of $Fe_{2.84\pm0.05}GaTe_2$. a Magnified ABF-STEM image of a single $Fe_{2.84\pm0.05}GaTe_2$ layer. **b** Integrated imaging intensity line profile along the *c*-axis within the area marked by the Te-Fe_{ii}-Te atoms in the left blue rectangle. The red region indicates the Fe_{ii} deviation from the centers of Te-Te atoms. **c** Integrated imaging intensity line profile of $Fe_{i\text{-}a}$ and $Fe_{i\text{-}b}$ atoms along the c-axis within the area indicated by the right rectangle.

Supplementary Note 2: Fig. S4 shows HAADF- and ABF-STEM images of the $Fe_{2.84\pm0.05}GaTe_2$ sample along the [11$\bar{2}$0] zone axis, which provide clear view of the Fe_i and Fe_{ii} columns. To highlight the detailed information about the Fe_{ii} columns, a magnified image was derived from the enclosed region of the ABF-STEM image, as shown in Fig. S5a. For a quantitative determination of the deviation of the Fe_{ii} atoms, we focused on the region marked by left the blue rectangle in Fig. S5a comprising Te-Fe_{ii}-Te atoms. We then vertically integrated the corresponding imaging intensity line profile (Fig. S5b). By referencing the center of the two Te atoms, the deviation of the Fe_{ii} atom towards the −*c* direction was determined to be −0.20 Å. Utilizing the same procedure, we surveyed an area of 2 × 17 unit cells, yielding an average Fe_{ii} deviation of −0.16 ± 0.06 Å.

Additionally, we observed that the image intensity of $Fe_{i\text{-}a}$ above Fe_{ii} is weaker than that of $Fe_{i\text{-}b}$ below Fe_{ii}, as evident in the imaging intensity line profile of $Fe_{i\text{-}a}$ and $Fe_{i\text{-}b}$ atoms in Fig. S5c. Since imaging intensity is generally proportional to the number of projected atoms[1], the contrast difference between $Fe_{i\text{-}a}$ and $Fe_{i\text{-}b}$ indicates asymmetric site occupations, suggesting a small quantity of Fe vacancies in the $Fe_{i\text{-}a}$ site.

6. XRD refined crystal structure and simulated HR-STEM and SAED images.

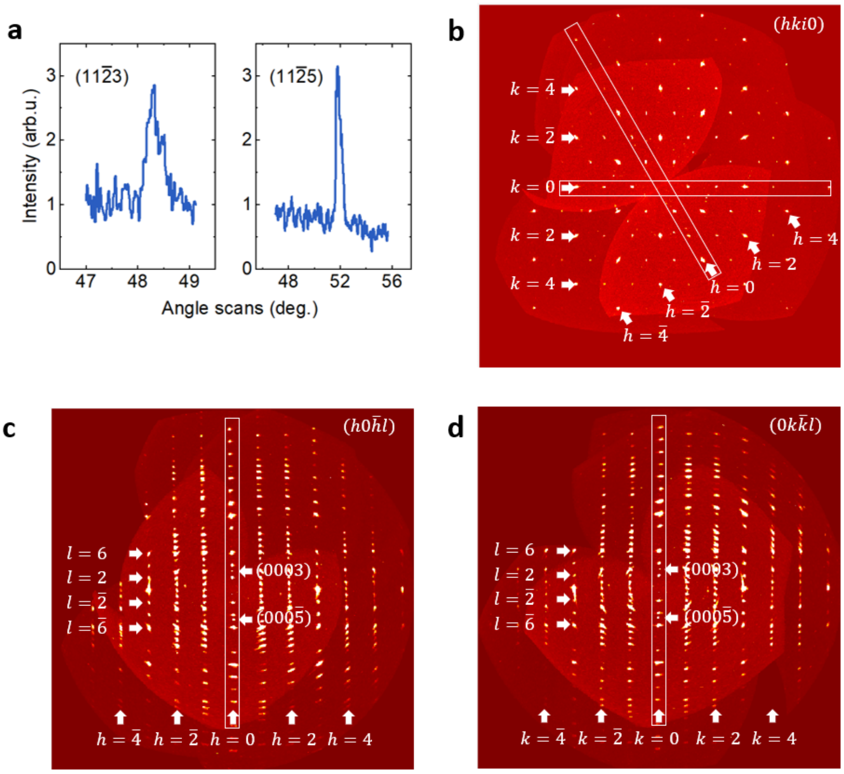

Fig. S6. | Single-crystal XRD characterizations. a XRD angle scans by four-circle diffractometer showing the typical reflections. Note that, the observed weak (11$\bar{2}$3) and (11$\bar{2}$5) reflections are forbidden for centrosymmetric space group $P6_3/mmc$. **b-d** The synthetic X-ray diffraction patterns of $Fe_{2.84\pm0.05}GaTe_2$ single crystal for **b** ($hki0$), **c** ($h0\bar{h}l$), and **d** ($0k\bar{k}l$) reflections. Note that, the observed (0003) and (000$\bar{5}$) reflections are forbidden for centrosymmetric space group $P63/mmc$.

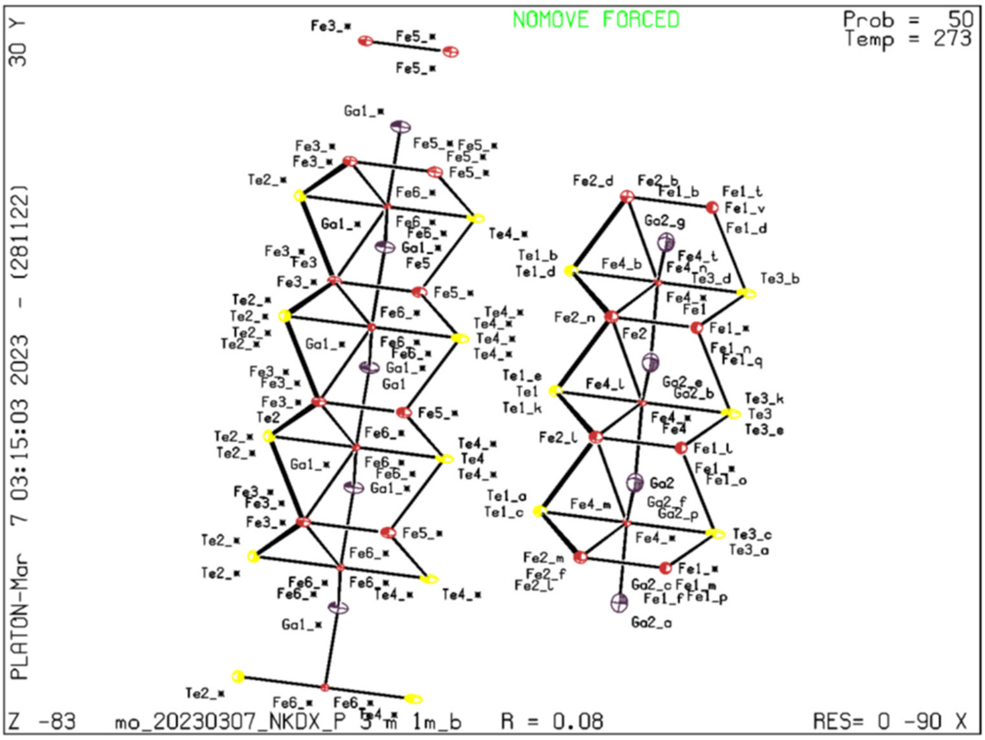

Fig. S7. | XRD refined crystal structure of $Fe_{2.84\pm0.05}GaTe_2$. The Fe_{ii} sites (indicated as Fe6 and Fe4) deviate from the center position of Te-Te atoms, and both offsets in the top and bottom layers are the same direction, indicating a non-centrosymmetric crystal structure.

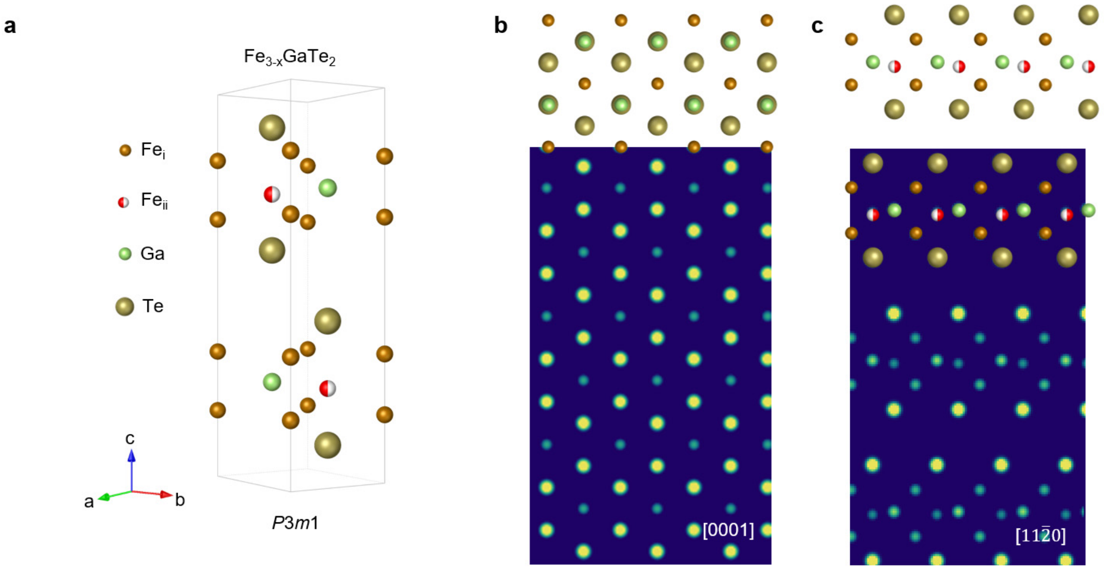

Fig. S8. | Schematic diagram of $Fe_{2.84\pm0.05}GaTe_2$ crystal structure. a Crystal structure of non-stoichiometric $Fe_{2.84\pm0.05}GaTe_2$ that is built with the refined Wyckoff site from XRD analysis. **b,c** Simulated HADDF images based on Dr. Probe [2], and the corresponding atom arrangements along the [0001] and [11$\bar{2}$0] zone axis.

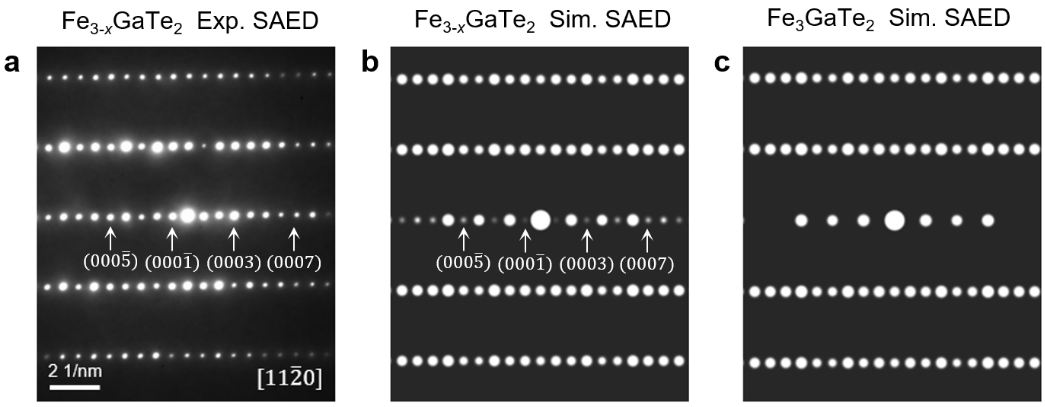

Fig. S9. **| Experimental and simulated $Fe_{3\text{-}x}GaTe_2$ SAED patterns. a** Selected Area Electron Diffraction (SAED) along the $[11\bar{2}0]$ zone axes. **b** Simulated SAED patterns based on the XRD refined non-centrosymmetric structure of $Fe_{3\text{-}x}GaTe_2$. **c** Simulated SAED patterns based on the prefect centrosymmetric structure of Fe_3GaTe_2.

Supplementary Note 2: A specimen of $Fe_{2.79}GaTe_2$ was used for the X-ray crystallographic analysis. The X-ray intensity data were measured (λ = 0.71073 Å). The frames were integrated with the Bruker SAINT software package using a narrow-frame algorithm. The integration of the data using a trigonal unit cell yielded a total of 1504 reflections to a maximum θ angle of 28.01° (0.76 Å resolution), of which 534 were independent (average redundancy 2.816, completeness = 99.3%, R_{int} = 5.18%, R_{sig} = 5.61%) and 455 (85.21%) were greater than $2\sigma(F^2)$. The final cell constants of a = 4.0804(6) Å, b = 4.0804(6) Å, c = 16.127(4) Å, volume = 232.54(9) Å^3, are based upon the refinement of the XYZ-centroids of 2600 reflections above 20 σ(I) with 5.052° < 2θ < 56.00°. Data were corrected for absorption effects using the Multiscan method (SADABS). The ratio of minimum to maximum apparent transmission was 0.393.

The structure was solved and refined using the Bruker SHELXTL Software Package, using the space group $P3m1$, with Z = 2 for the formula unit, $Fe_{2.79}GaTe_2$. The final anisotropic full-matrix least-squares refinement on F^2 with 38 variables converged at $R1$ = 8.33%, for the observed data and $wR2$ = 25.87% for all data. The goodness-of-fit was 1.192.

Table S2. Refined crystal data for $Fe_{3-x}GaTe_2$.

**Chemical formula**	$Fe_{2.79}GaTe_2$	
**Formula weight**	480.80 g/mol	
**Temperature**	273(2) K	
**Crystal system**	trigonal	
**Space group**	*P3m1*	
**Unit cell dimensions**	a = 4.0804(6) Å	α = 90°
	b = 4.0804(6) Å	β = 90°
	c = 16.127(4) Å	γ = 120°
**Volume**	232.54(9) Å^3	
**Density (calculated)**	6.867 g/cm^3	

7. Structural relaxation and electron density for Fe vacancies model.

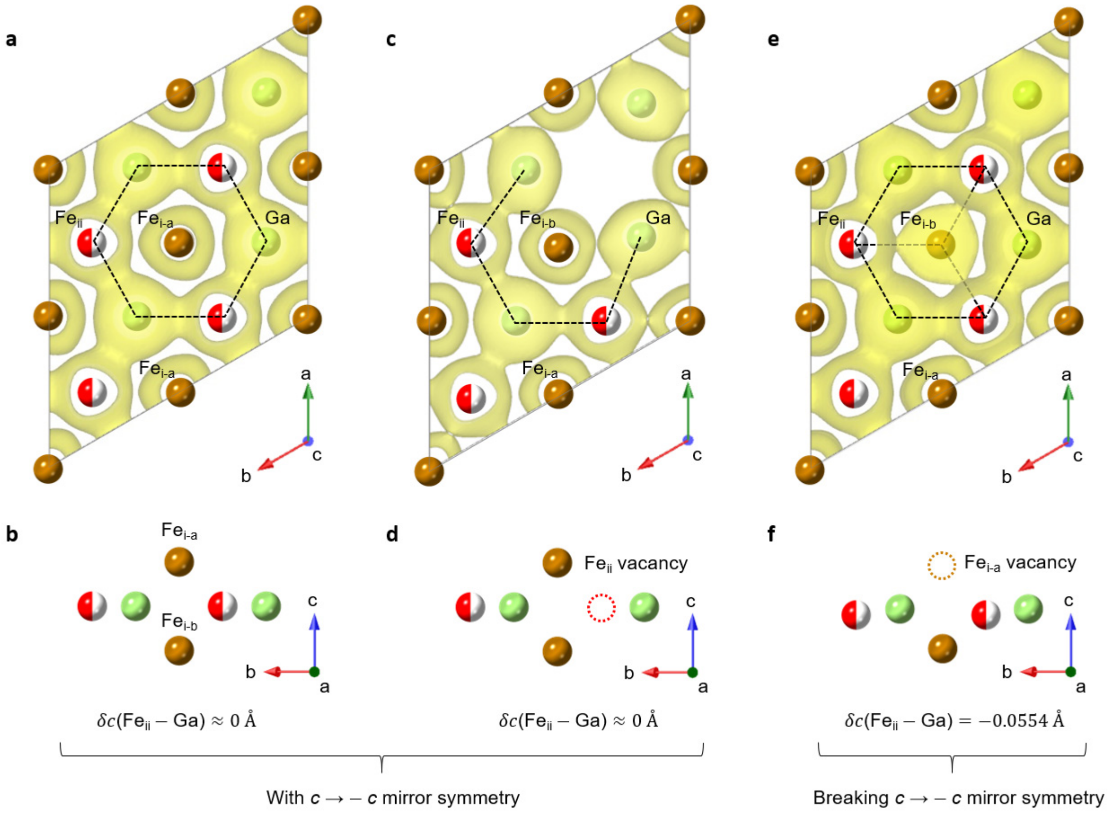

Fig. S10. | First-principles calculations for Fe vacancies model. The relaxed crystal structure and corresponding electron density of $Fe_{3-x}Ga$ atoms sliced from $Fe_{3-x}GaTe_2$ with **a**, **b** no vacancy, **c**, **d** Fe_{ii} vacancy and **e**, **f** $Fe_{i\text{-}a}$ vacancy. The black dashed line indicates the chemical bonding with electron-density overlapping. The yellow-colored electron densities are shown at the same isosurface value.

Supplementary Note 3: Having established the crystal structure, we conducted first-principles calculations to analyze the impact of $Fe_{i\text{-}a}$ and Fe_{ii} vacancies on Fe_{ii} deviation. Starting with a perfect centrosymmetric Fe_3GaTe_2 lattice, we constructed a 2×2 supercell encompassing a total of four molecular layers. Within the bottommost layer, we systematically introduced three scenarios: no vacancy, $Fe_{i\text{-}a}$ vacancy, and Fe_{ii} vacancy. Lattice relaxations were performed independently for the three distinct supercells. In order to illustrate the Fe vacancies and further compare the alterations in Fe_{ii} chemical bonding, we presented the electron density of $Fe_{3-x}Ga$ atoms within the bottommost layer (Fig. S10).

Fig. S10a and S10b illustrate the scenario with no vacancy. The electron density

(colored in yellow) strongly overlaps between Fe_{ii}-Ga atoms, forming the Fe_{ii}-Ga honeycomb lattice plane with robust chemical bonding (highlighted by black dashed lines). Simultaneously, the $Fe_{i\text{-}a}$ and $Fe_{i\text{-}b}$ dimers are located at the center of the Fe_{ii}-Ga honeycomb lattice but do not bond with Fe_{ii}-Ga atoms. Consequently, the chemical bonding is mirror-symmetric along the Fe_{ii}-Ga plane, leading to the absence of Fe_{ii} displacements. Thus, perfect Fe_3GaTe_2 exhibits a centrosymmetric crystal structure with $c \rightarrow -c$ mirror symmetry.

Fig. S10c and S10d illustrate the scenario with Fe_{ii} vacancy. Although Fe_{ii} vacancies cause deformation of the Ga electron density within the *ab* plane, there is still no overlapping with of $Fe_{i\text{-}a}$ and $Fe_{i\text{-}b}$ in the *c* direction. This observation indicates that the remaining Fe_{ii} primarily forms bonds with Ga in the *ab* plane, with no displacement observed in the *c* direction.

Fig. S10e and S10f illustrate the scenario with $Fe_{i\text{-}a}$ vacancy. Apart from the strongly bonded Fe_{ii}-Ga honeycomb lattice, there is additional electron-density overlapping among the lower $Fe_{i\text{-}b}$ atom and its three nearest Fe_{ii} atoms, while no overlapping between $Fe_{i\text{-}b}$ and its three nearest Ga atoms. Consequently, the newly formed chemical bonding (highlighted by black dashed line) will drag the Fe_{ii} atoms displacing downwards to the lower $Fe_{i\text{-}b}$ atom. Notably, the determined deviation between nearest Fe_{ii} and Ga atoms in the relaxed structure is –0.0554 Å. This value aligns with the analysis from ABF-STEM image, confirming the reliability of our DFT model. Based on the DFT results above, we conclude that the asymmetric vacancy of $Fe_{i\text{-}a}$ induces a displacement of Fe_{ii} atoms towards the $-c$ direction.

In addition, we calculated the vacancy formation energy for Fe_i and Fe_{ii}. As for the calculation, we used a structure that repeats 3 times in the *c* direction and removed the bottommost $Fe_{i\text{-}a}$ or Fe_{ii} atoms, and compared its total free energy with the parent structure. The calculated values of formation energy for Fe_i and Fe_{ii} was 2.96 eV/Fe and 2.86 eV/Fe, respectively. This result suggests that Fe_{ii} vacancies are more likely to occur, aligning with experimental results.

Specific computational details: In all cases presented, the Vienna ab initio simulation package (VASP) was used with electron-core interactions described by the projector augmented wave method for the pseudopotentials, and the exchange correlation energy calculated with the generalized gradient approximation of the Perdew-Burke-Ernzerhof (PBE) form. The plane wave cutoff energy was 400 eV for all the calculations. In calculating the atomic shifts due to Fe_{i-a} and Fe_{ii} vacancies, we used a 2×2 supercell and removed the bottommost Fe_{i-a} and Fe_{ii} atoms. The Monckhorst-Pack scheme was used for the Γ-centred 12 × 12 × 1 k-point sampling. All atoms' relaxations were performed until the force become smaller than 0.001 eV/A for determining the most stable geometries.

8. First-principles calculations for Dzyaloshinskii-Moriya interaction.

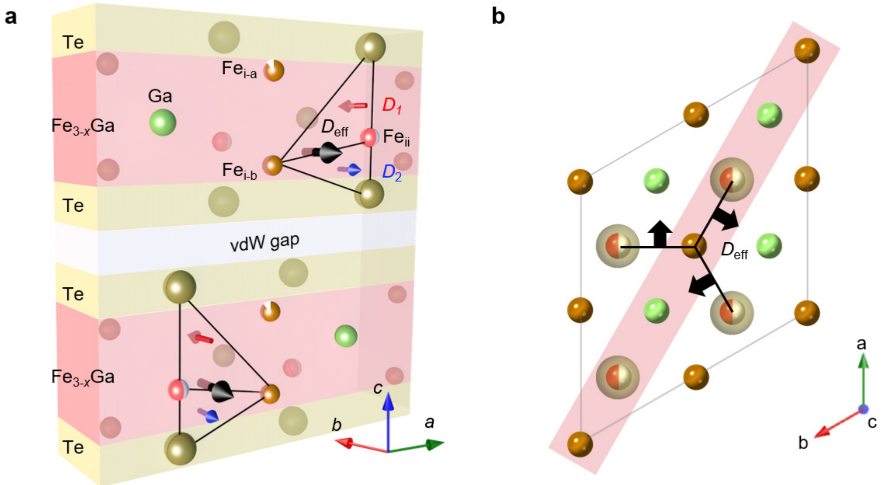

Fig. S11. | Schematic illustration of DMI. a Schematic illustration of DMI in asymmetric layers viewed from $[11\bar{2}0]$ zone axis. The red arrow D_1 represents the direction of DMI vector in the upper triangle composed of Fe_i-Fe_{ii}-Te, while the blue arrow D_2 represents the lower part in the opposite direction. The black arrow D_{eff} represents the sum of the non-zero DMI vector. **b** Schematic illustration of DMI viewed from [0001] zone axis. The black arrow represents the effective net DMI vectors D_{eff}. And the red rectangular represents the slice of atoms in the left panel **a**.

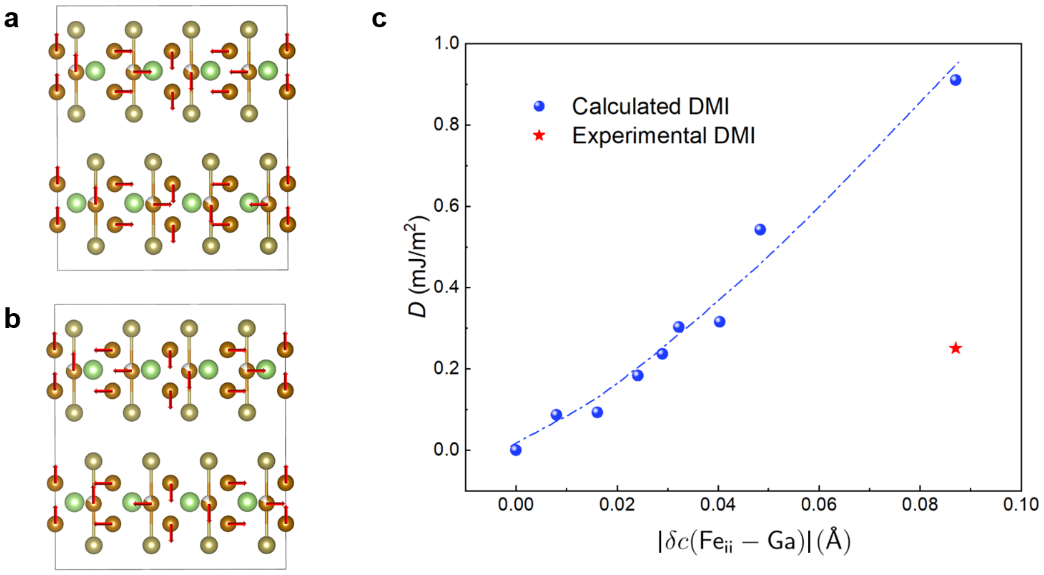

Fig. S12. | First-principles calculations of DMI. a, b Spin configurations implemented to calculate the DMI for clockwise (CW) and anticlockwise (ACW). **c** The calculated and experimental results for the relationship between the DMI and the Fe_{ii} deviation.

Supplementary Note 4: In order to compare the DFT calculations with experimental observations, we investigated the relationship between Fe_{ii} deviation value $\delta c(Fe_{ii} - Ga)$ and DMI constant *D*. As shown in Fig. S11 and S12, the crystal structures were built with different Fe_{ii} deviation values $\delta c(Fe_{ii} - Ga)$, which are fixed during the two-step calculation for DMI values. The calculated *D* with different $\delta c(Fe_{ii} - Ga)$ values are shown in Fig. S12c. On the one hand, the minimum $\delta c = 0$ indicate a centrosymmetric structure without Fe_{ii} deviation. And the corresponding DMI value is $D = 0$ mJ/m^2, which is consistent with our expectation. On the other hand, the maximum $\delta c = -0.0871$ Å is equaling to the non-centrosymmetric structure determined by single-crystal XRD. And the corresponding DMI value is $D = 0.91$ mJ/m^2. Based on the measured magnetic domain width from LTEM experiments, the extracted $D = 0.25$ mJ/m^2 falls within the range of DFT calculated *D* values.

Specific computational details: All calculations are based on the Vienna *ab initio* simulation package with electron-core interactions described by the projector augmented wave method for the Perdew-Burke-Ernzerhof (PBE) form[3-5], and the plane-wave cutoff energy is set to 400 eV. The 4 × 16 × 4 k-point grids were used to sample the Brillouin zones for the 4 × 1 × 2 Fe_3GaTe_2 supercell. To obtain the DMI vector, calculations were performed in two steps. First, structural relaxations were performed with Gaussian smearing until the forces become smaller than 0.001 eV/Å. Next, spin-orbit coupling was included in the calculation, and the total energy of the system was determined as a function of the spin configuration as shown in Fig. S12a, and $d_{||}$ equals to $(E_{ACW} - E_{CW})/12$. The DMI constant *D* was calculated using the equation $D = 3\sqrt{2}d/(N_F a^2)$, where N_F is the number of atomic layers, *a* is the lattice constant and $d_{||}$ represents DMI strength[3]. In the second step, the EDIFF is set to 10^{-8} eV and the tetrahedron method with Blöchl corrections was used to get an accurate total-energy. This method has been widely used in DMI calculations including bulk frustrated system and insulating chiral-lattice magnets[3-6].

9. Thickness dependent Hall resistivity at room-temperature.

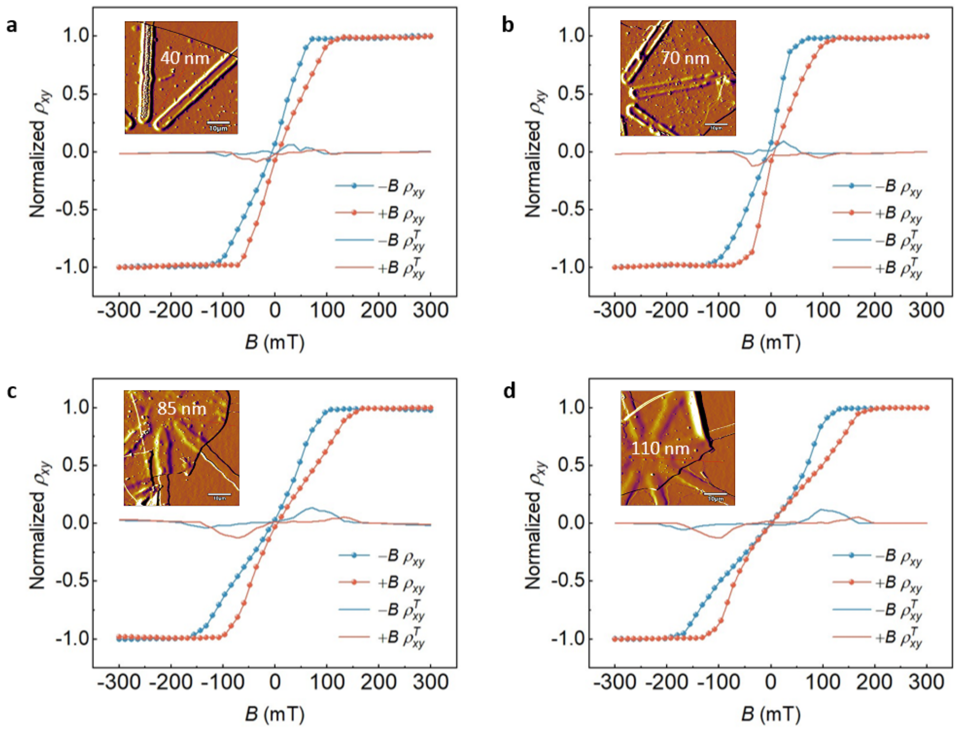

Fig. S13. | Thickness dependent Hall measurements. a-d Room-temperature magnetic hysteresis of Hall resistivity ρ_{xy} and topological Hall resistivity ρ_{xy}^{T} at various sample thickness from 40 nm to 110 nm. Red (blue) curves were measured with increasing (decreasing) magnetic field. The insets show the optical images of the $Fe_{2.84\pm0.05}GaTe_2$ Hall devices.

10. Detailed Lorentz phase images and AFM images.

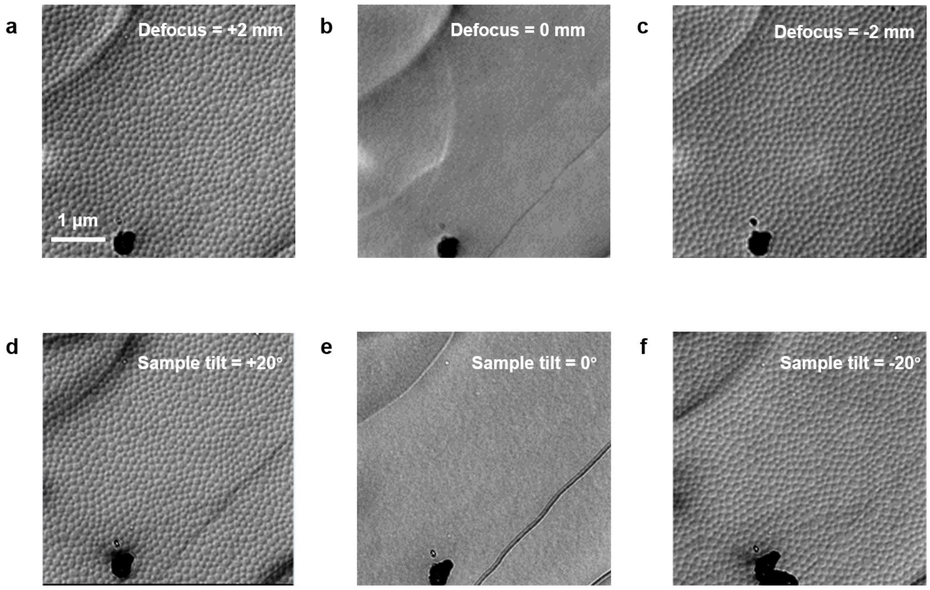

Fig. S14. | Detailed LTEM characterizations. a Over-focused, **b** in-focus, and **c** under-focused Lorentz phase images for room-temperature (RT) skyrmions after field-cooling procedure (30 mT). **d** +20°, **e** 0° and **f** −20°sample tilt of Lorentz phase images for RT Néel-type skyrmions. These images were taken at a defocus value of +2 mm.

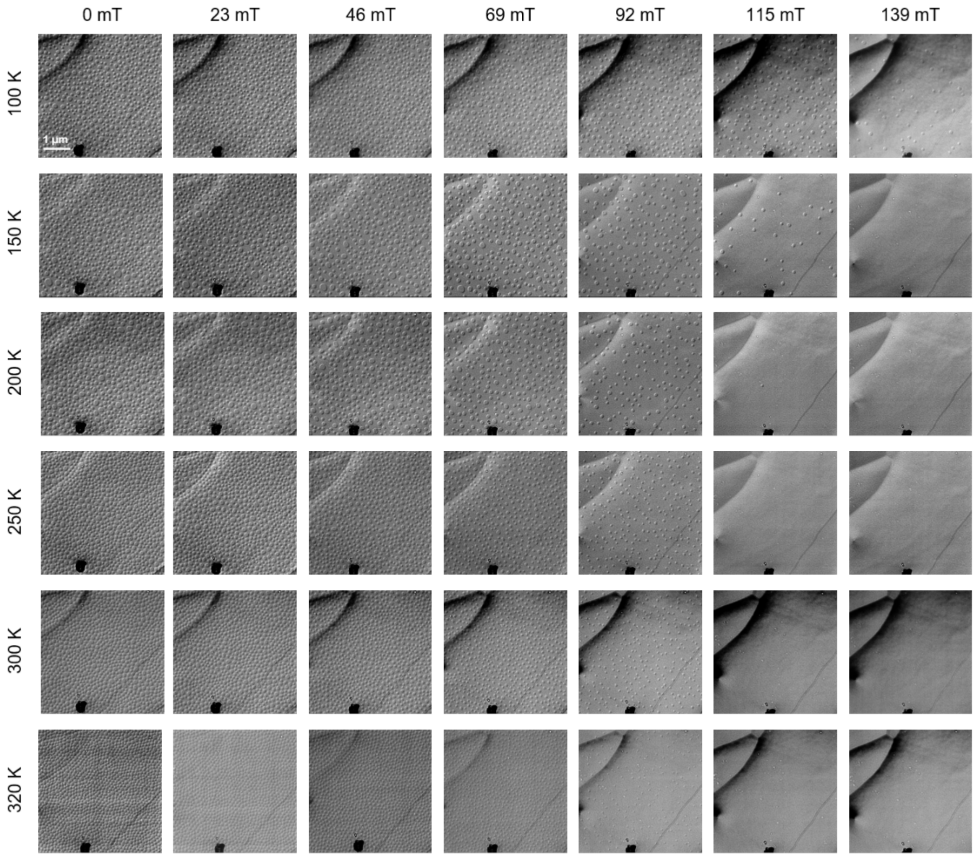

Fig. S15. | Temperature and magnetic field dependent LTEM characterizations. Series of Lorentz phase images taken at various magnetic fields and temperatures with a defocus value of +2 mm and +20° sample tilt.

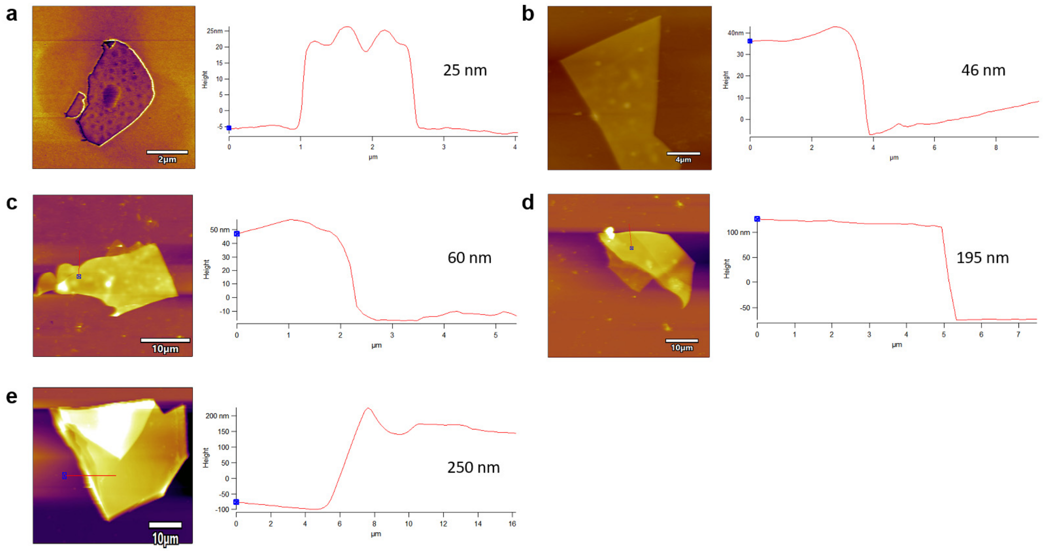

Fig. S16. | AFM measurements. a-e Sample morphology and thickness analysis by Atomic Force Microscope (AFM).

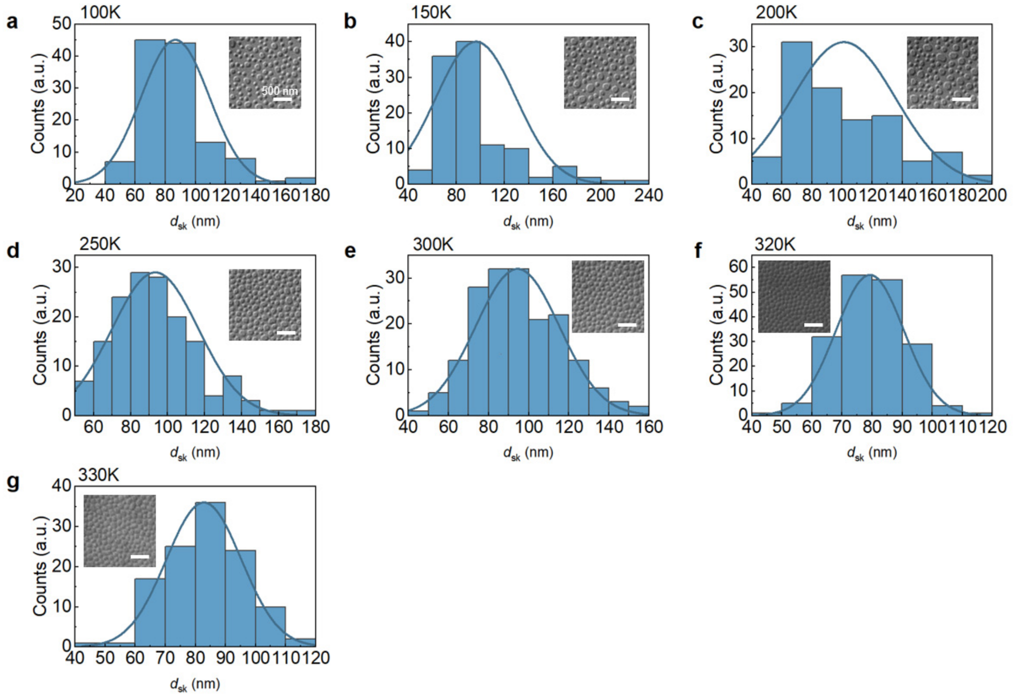

Fig. S17. | Skyrmion size distribution. a-g Statistics on the size distribution of field-free skyrmions at various temperatures.

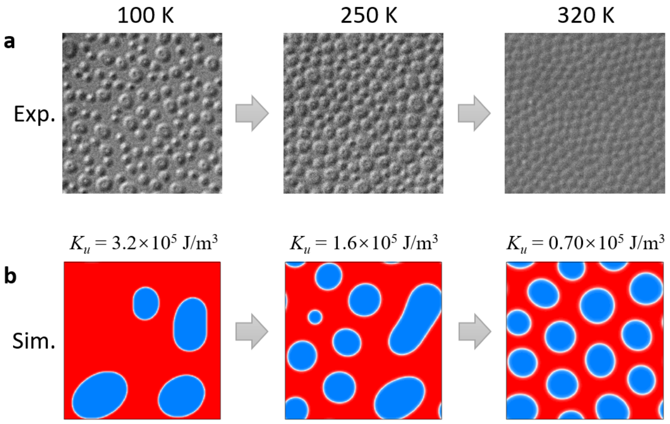

Fig. S18. | Simulated skyrmion size distribution. a Lorentz Phase images of zero-field skyrmions after 30 mT field cooling at 100 K, 250 K and 320 K. **b** Micromagnetic simulations of zero-field skyrmions after 30 mT field cooling with magnetic anisotropy constant $K_u = 3.2 \times 10^5$ J/m^3, 1.6×10^5 J/m^3 and 0.7×10^5 J/m^3.

Supplementary Note 4: In the LTEM experiments, we initially raised the sample temperature to above the Curie temperature, then cooled it to the target temperature with a 30 mT external magnetic field, and removed the external magnetic field to obtain the zero-field skyrmions. As shown in Fig. S17 and S18, the density of the zero-field skyrmions at low temperature (100 K) is relatively low, exhibiting non-uniformed size distribution. However, at higher temperatures of 250 K and 320 K, the zero-field skyrmion density gradually increases, and the size distribution becomes more uniform.

To clarify physical mechanism underlying the variation in skyrmion size at different temperatures, we conducted micromagnetic simulations of zero-field skyrmions after field cooling. As is known, the formation of skyrmions is determined by the magnetic parameters, such as magnetic anisotropy K_u, DMI constant D, saturation magnetization M_s, sample thickness t, and exchange stiffness A. However, increasing the sample temperature of $Fe_{3-x}GaTe_2$ results in the most notable variations in the reduction of magnetic anisotropy K_u. Therefore, as shown in Fig. S18, we simulated zero-field skyrmion after 30 mT field cooling with magnetic anisotropy constant $K_u = 3.2 \times 10^5$ J/m^3, 1.6×10^5 J/m^3 and 0.7×10^5 J/m^3, which is in accordance with the increasing of sample temperature. Our simulations demonstrate that with large K_u at low temperature, the density of zero-field skyrmions is low. And the distant between the nearest skyrmions can be considerably large in certain regions, thus facilitating the expansion of skyrmion size upon the removal of the magnetic field. In contrast, with small K_u at high temperature, the skyrmions exhibit a densely hexagonal arrangement to each other, which suppresses the extension of the skyrmions. Consequently, they remain uniformly distributed after removing the magnetic field.

Details about the simulation: To validate this experimental results, we conducted micromagnetic simulations with 30 mT field cooling process and then removing the external magnetic field. Default magnetic parameters used in the simulations include $A = 1.3$ pJ/m, $K_u = 0.8 \times 10^5$ J/m^3, $M_s = 2.5 \times 10^5$ A/m, $D = 0.25$ mJ/m^2, and slab geometries with dimensions of $512 \times 512 \times 64$, with a mesh size of $2 \times 2 \times 2$ nm. Periodic boundary conditions were taken into account for large-scale simulations.

11. Determination of magnetic parameters for $Fe_{2.84\pm0.05}GaTe_2$.

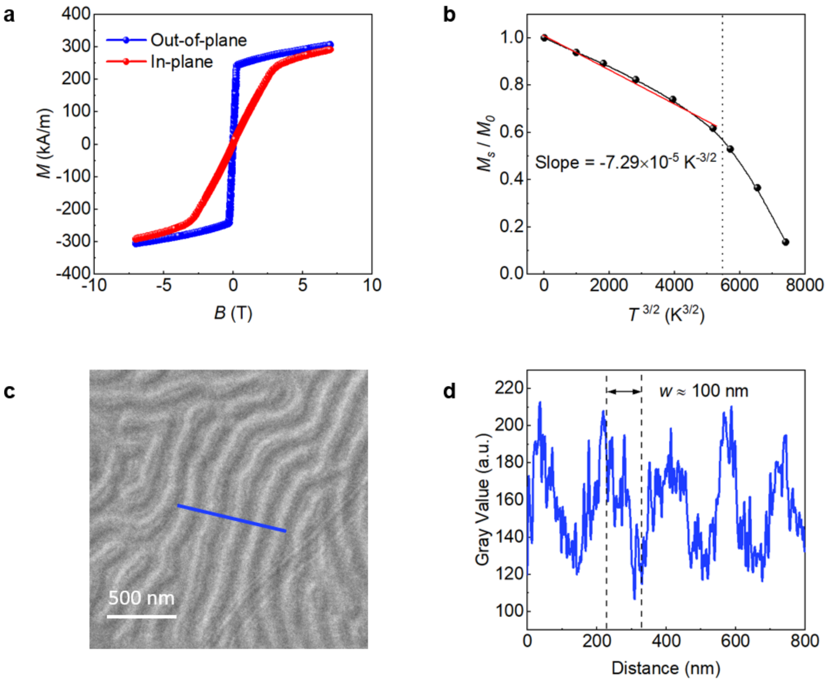

Fig. S19. | Magnetic characterizations of $Fe_{2.84\pm0.05}GaTe_2$. a Field dependence of magnetization at room temperature in bulk single crystal. **b** Normalized saturation magnetization M_s/M_0 versus $T^{3/2}$. The red line indicates the linear fitting. **c** Stripe domains at room temperature. **d** The corresponding gray value indicates domain wall period $w \approx 100$ nm.

Supplementary Note 5: Micromagnetic simulations were carried out using the GPU-accelerated micromagnetic simulation program Mumax[3]. The slab geometries of dimensions were 512 × 512 × 64 nm with a mesh size of 2 × 2 × 2 nm. Periodic boundary conditions were taken into account for large-scale simulations. The total energy of the micromagnetic model contained exchange interactions, first-order uniaxial anisotropy, antisymmetric Dzyaloshinskii-Moriya interactions, magnetostatic energy and Zeeman energy.

In these simulations, the input parameters were determined from experimental measurements. As shown in Fig. S19a, the perpendicular magnetic anisotropy favors out-of-plane magnetization. We can thus estimate the saturation magnetization $M_s = 2.5 \times 10^5$ A/m. We consider the anisotropy constant in thin nanoflakes to be $K_u = 0.8 \times 10^5$ J/m^3.

The exchange stiffness A was determined by fitting the temperature dependence of magnetization with Bloch's $T^{3/2}$ law[7] (Fig. S19b):

$$M_s(T) = M_0\left[1 - 0.0586\frac{g\mu_B}{M_0}\left(\frac{kT}{d}\right)^{3/2}\right], \tag{1}$$

where M_0 is the saturation magnetization, g is the Landé g-factor, μ_B is the Bohr magneton, k is the Boltzmann constant, and d is the spin-wave stiffness that given by:

$$A = \frac{M_0}{2g\mu_B}d. \tag{2}$$

Therefore, we can extract the exchange stiffness A = 1.3 pJ/m.

The value of DMI constant D can be quantified by estimating the domain wall energy:

$$\sigma_{DW} = 4\sqrt{AK} - \pi|D|, \tag{3}$$

where σ_{DW} is the domain wall surface energy density, which can be calculated by measuring the domain wall period w on the basis of a domain spacing model[8]:

$$\frac{\sigma_{DW}}{\mu_0 M_s^2 t} = \frac{w^2}{t^2}\sum_{odd\ n=1}^{\infty}\left(\frac{1}{(\pi n)^3}\right)[1 - (1 - 2\pi nt/w)\exp(-2\pi nt/w)], \tag{4}$$

where t is the sample thickness. As shown in Figs. S19c and S19d, the domain wall period w is approximately 100 nm. With a sample thickness t = 60 nm, we can calculate the DMI constant D = 0.25 mJ/m^2.

12. Micromagnetic simulations for skyrmion sizes.

Table S3. Magnetic parameters for skyrmion-host 2D materials.

	$(Fe_{0.5}Co_{0.5})_5GeTe_2$	$Fe_{3-x}GaTe_2$
D (mJ/m^2)	0.90	0.25
M_s (× 10^5 A/m)	3.0	2.5
t (nm)	≥110	≥46
K_u (× 10^5 J/m^3)	2.4	0.8
A (pJ/m)	4.0	1.3
T (K)	300	300

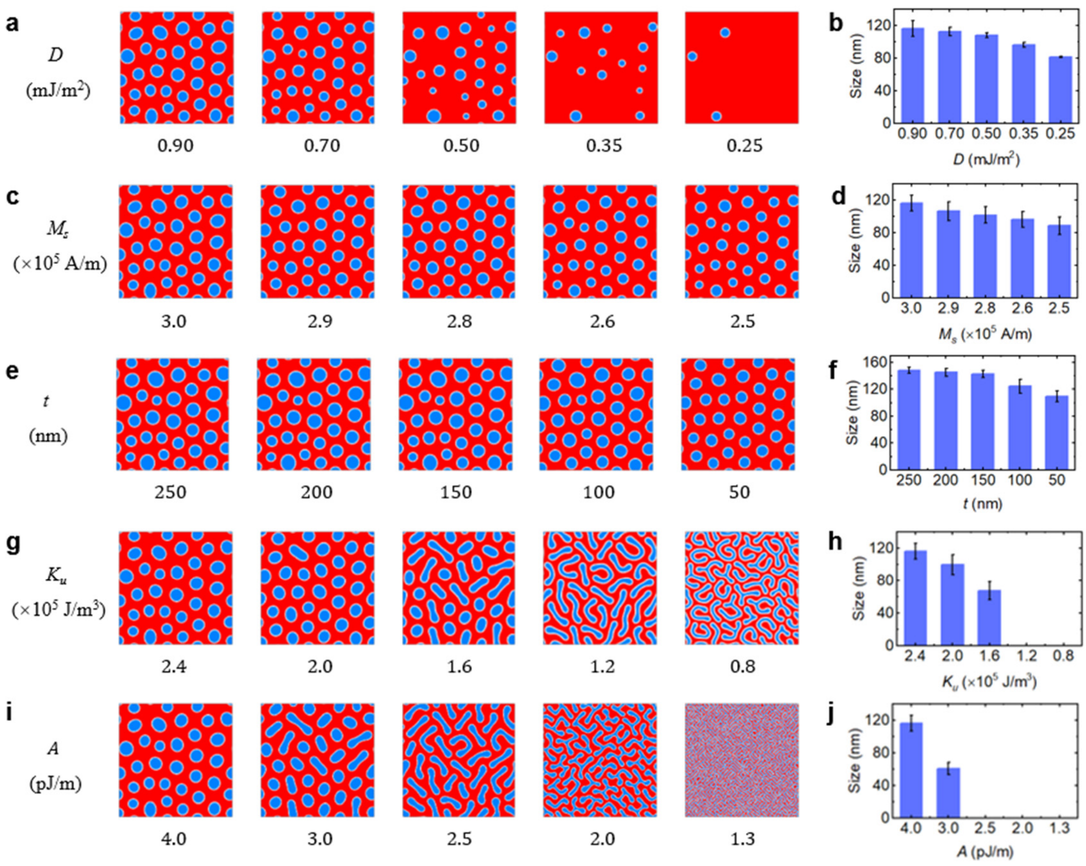

Fig. S20. | Micromagnetic simulations for skyrmion sizes. Variation of simulated magnetic structure and corresponding skyrmion sizes with varied DMI constant D (**a**, **b**), saturation magnetization M_s (**c**, **d**) , sample thickness t (**e**, **f**), magnetic anisotropy constant K_u (**g**, **h**) , and exchange stiffness A (**i**, **j**).

Supplementary Note 6: It is widely recognized that the skyrmion size is greatly influenced by the interplay among various factors, including DMI, magnetic dipolar interaction, magnetic exchange interaction, saturation magnetization, and magnetic anisotropy. Utilizing the experimentally established magnetic parameters associated with these magnetic interactions in different vdW magnets (Table S3), we conducted micromagnetic simulations to clarify the contributions of these factors to the skyrmion size.

In our initial simulation, we modeled the zero-field skyrmion state following a 60 mT field cooling, adopting the magnetic parameters of $(Fe_{0.5}Co_{0.5})_5GeTe_2$ as a reference[9]. The simulation results, as depicted in Fig. S20, revealed a high-density of skyrmions with an average size of approximately 116 nm. Subsequently, by initiating the simulation with this skyrmion state and progressively reducing the DMI constant D to match that of $Fe_{3-x}GaTe_2$ while keeping other magnetic parameters constant (Fig. S20a and S20b), we observed a significant decrease in the skyrmion size to 81 nm at D = 0.25 mJ/m^2. Employing a similar approach, we further simulated the magnetic domain states by varying the saturation magnetization M_s, sample thickness t, magnetic anisotropy constant K_u and exchange stiffness A towards those of $Fe_{3-x}GaTe_2$. It is clearly demonstrated that each parameter reduction leads to a decrement in the skyrmion size. Thus, our simulations suggest that smaller magnetic parameters such as D, M_s, t, K_u, and A in $Fe_{3-x}GaTe_2$ (in comparison to $(Fe_{0.5}Co_{0.5})_5GeTe_2$), contribute to the reduction in skyrmion size.

Details about the micromagnetic simulation: Micromagnetic simulations were carried out using the GPU-accelerated micromagnetic simulation program Mumax^3. Unless specified otherwise, default magnetic parameters used in the simulations include A = 4.0 pJ/m, $K_u = 2.4 \times 10^5$ J/m^3, $M_s = 3.0 \times 10^5$ A/m, D = 0.90 mJ/m^2, and slab geometries with dimensions of 512 × 512 × 64, with a mesh size of 2 × 2 × 2 nm. Periodic boundary conditions were taken into account for large-scale simulations. The initial skyrmion state was relaxed from a random state with 60 mT magnetic field. Employing the initial skyrmion state as the input, we systematically varied the magnetic

parameters—D, M_s, t, K_u, and A individually—subsequently allowing the magnetization to evolve into a stabilized state through relaxation processes.

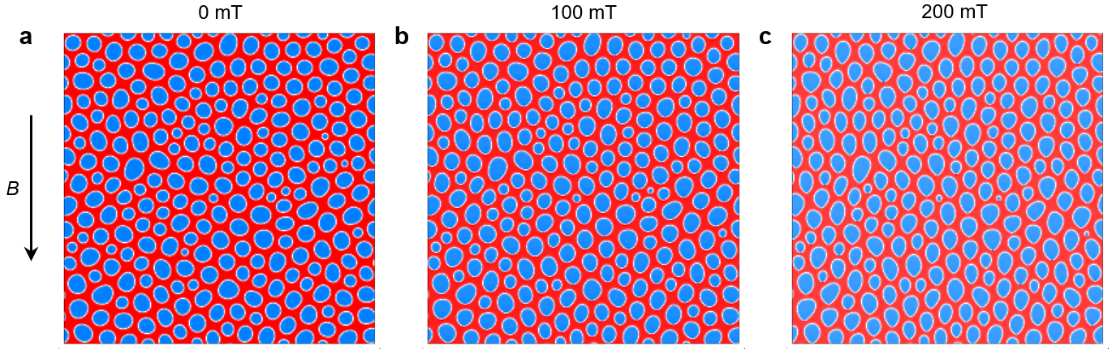

Fig. S21. | Micromagnetic simulations for skyrmion shapes. Micromagnetic simulations of skyrmion evaluation with in-plane magnetic field B at **a** 0 mT, **b** 100 mT and **c** 200 mT, respectively. The shape of skyrmion transforms gradually from circular to elliptical.

Supplementary Note 7: As observed in our previous LTEM and MFM experiments (Fig. 3 and Fig. 4 in the main text), the out-of-plane magnetic field B did not change the shape of skyrmions. The skyrmions remained circular and exhibited a reduction in size as increasing out-of-plane B. In order to further investigate the influence of skyrmion shape with in-plane magnetic field B, we performed micromagnetic simulations and the results are shown in Fig. S21. It can be seen that as the in-plane magnetic field increases, skyrmions transforms from circular to elliptical at $B = 200$ mT, with the elongated shape along the direction of the in-plane magnetic field.

Details about the simulation: Micromagnetic simulations were carried out using the GPU-accelerated micromagnetic simulation program Mumax^3. Default magnetic parameters used in the simulations include $A = 1.3$ pJ/m, $K_u = 0.8 \times 10^5$ J/m^3, $M_s = 2.5 \times 10^5$ A/m, $D = 0.25$ mJ/m^2, and slab geometries with dimensions of 1024 × 1024 × 16, with a mesh size of 2 × 2 × 4 nm. Periodic boundary conditions were taken into account for large-scale simulations. The initial zero-field skyrmion state was relaxed from a random state with 100 mT magnetic field. Employing the initial skyrmion state as the input, we systematically applied an in-plane magnetic field along y axis, and relaxed the magnetization to a stable state.

4 Calculation of two temperature model.

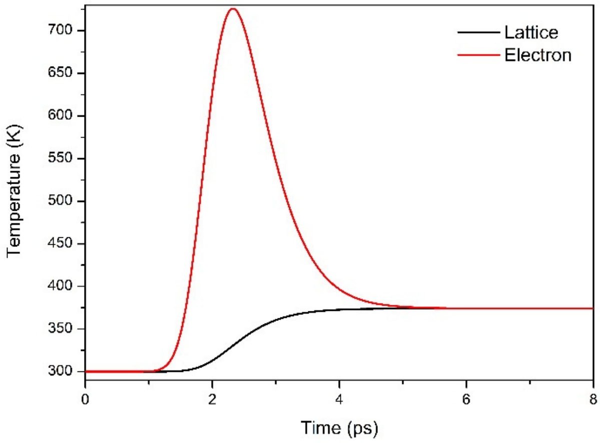

Fig. S22. | Two-temperature model. The simulated electron and phonon temperature versus delay times of $Fe_{2.84\pm0.05}GaTe_2$.

Supplementary Note 8: A phenomenological two-temperature model (2TM) model was utilized to describe the ultra-fast demagnetization dynamics during the fs-laser excitation on $Fe_{2.84\pm0.05}GaTe_2$. The 2TM includes the electron temperature T_{elec} and phonon (lattice) temperature T_{phon}, which can be described by the following equations:

$$C_e \frac{\partial T_{elec}}{\partial t} = -G_{ep}(T_{elec} - T_{phon}) + S(t), \quad (5)$$

$$C_p \frac{\partial T_{elec}}{\partial t} = -G_{ep}(T_{elec} - T_{phon}), \quad (6)$$

where C_e and C_p donate the heart capacities of the electron and phonon systems that independent of the temperature, G_{ep} is the electron-phonon coupling parameter, and $S(t)$ is the power of the single-shot laser pulse. The best fit variables of 2TM for $Fe_{2.84\pm0.05}GaTe_2$ are referred to the data of Fe_3GeTe_2 [10].

Based on Landau-Lifshitz-Gilbert equation, these initial field-polarized stripe domain state was relaxed by varying the out-of-plane magnetic field. As indicated by 2TM model (Fig. S22), the laser writing of skyrmion is dominated by thermal effect similar to that of field-cooling process. Therefore, we took a random spin configuration as the laser-melted spin state, and the subsequent skyrmion states after laser were relaxed with applying out-of-plane magnetic field. Furthermore, a damping constant α = 0.5 was used to quickly reach the equilibrium magnetic state.

5 Detailed in-situ optical LTEM experiments.

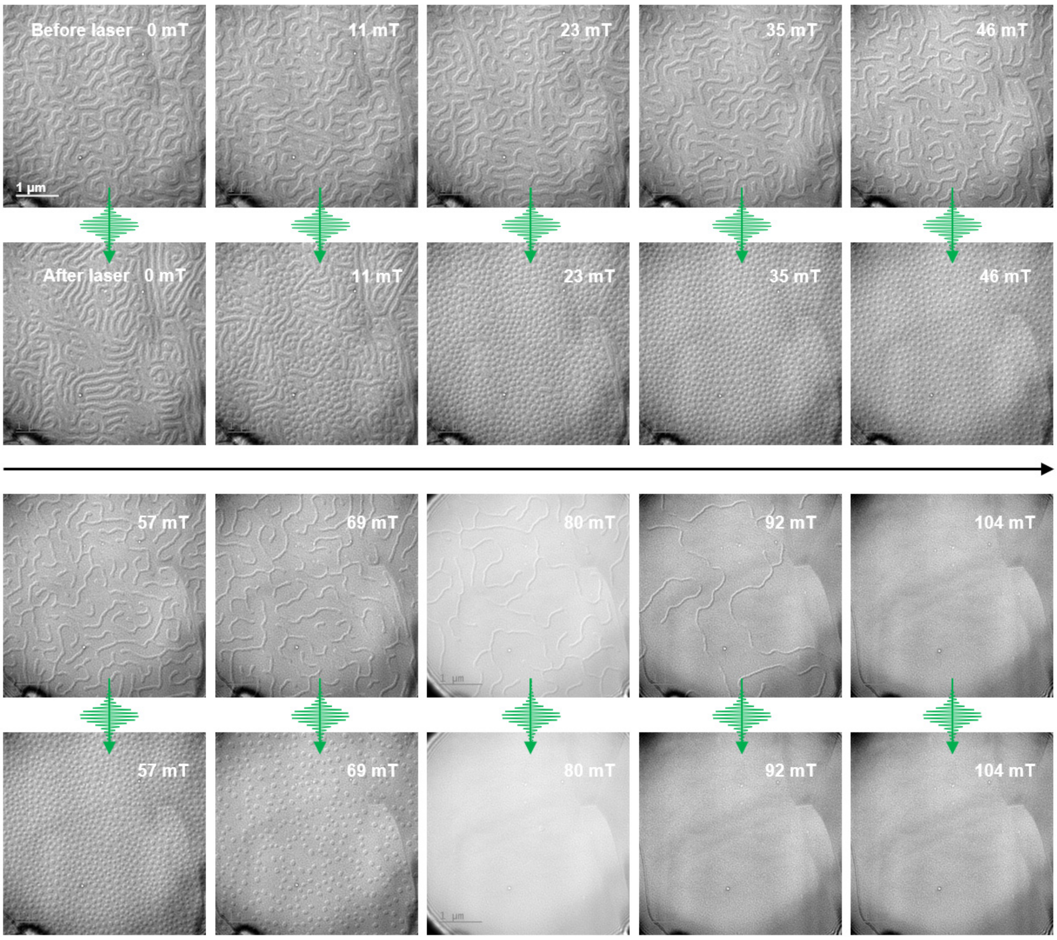

Fig. S23. | In-situ laser writing of skyrmions with increasing *B*. Step-by-step single-shot laser pulse excitation (11 mJ/cm^2) on a specific field-polarized magnetic state to determine the subsequent laser-accessible magnetic states. These images were recorded with increasing magnetic field.

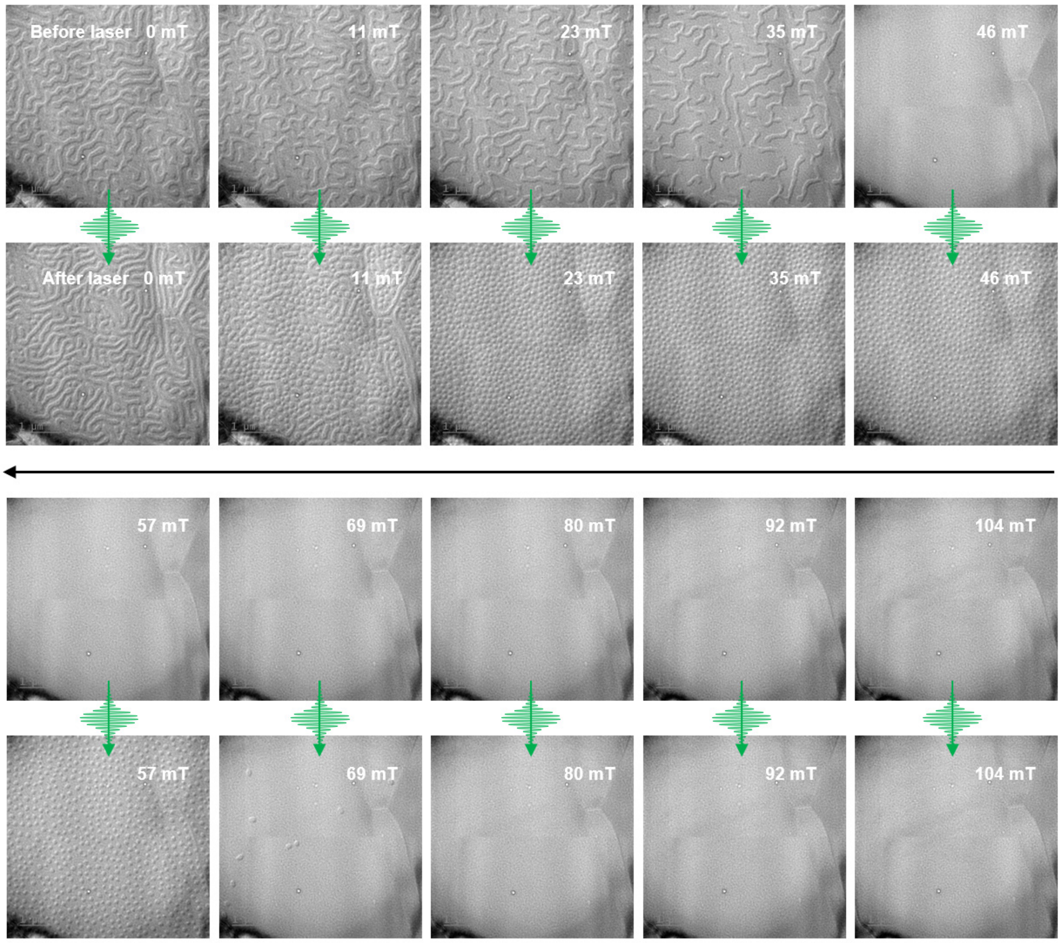

Fig. S24. | In-situ laser writing of skyrmions with decreasing *B*. Step-by-step single-shot laser pulse excitation (11 mJ/cm^2) on a specific field-polarized magnetic state to determine the subsequent laser-accessible magnetic states. These images were recorded with decreasing magnetic field.

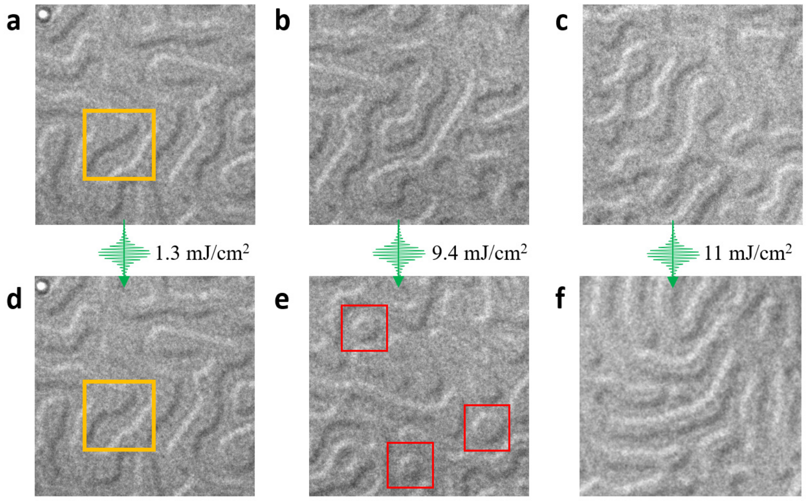

Fig. S25. | Fluence dependent in-situ laser writing of skyrmions. a, b, c. Ground states of the stripe domains obtained through zero-field cooling. **d, e, f.** Magnetic domain states after a single fs laser pulse with the fluence of 1.3 mJ/cm^2, 9.4 mJ/cm^2, and 11 mJ/cm^2, respectively. The red boxes indicated isolated skyrmions after a single fs laser pulse.

Supplementary Note 9: As depicted in Fig. S25a and S25d, under the condition of a single laser pulse fluence of 1.3 mJ/cm^2, the stripe domains within the yellow box merely exhibit domain wall movement after the laser pulse. In Fig. S25b and S25e, when we increase the single laser pulse fluence to 9.4 mJ/cm^2, the stripe domains become narrower and shorter, with some regions breaking to form skyrmions (highlighted in the red box). However, as we increase the single laser pulse fluence to 11 mJ/cm^2, stripe domains are formed without skyrmions (Fig. S25c and S25f). These in-situ laser fluence-dependent experiments indicate that a hybrid state with coexisting stripes and skyrmions is achievable without magnetic field.

References

1 Kourkoutis, L. F., Song, J. H., Hwang, H. Y. & Muller, D. A. Microscopic origins for stabilizing room-temperature ferromagnetism in ultrathin manganite layers. *Proceedings of the National Academy of Sciences* **107**, 11682-11685 (2010). https://doi.org:10.1073/pnas.1005693107

2 Barthel, J. Dr. Probe: A software for high-resolution STEM image simulation. *Ultramicroscopy* **193**, 1-11 (2018). https://doi.org:https://doi.org/10.1016/j.ultramic.2018.06.003

3 Yang, H., Thiaville, A., Rohart, S., Fert, A. & Chshiev, M. Anatomy of Dzyaloshinskii-Moriya Interaction at Co/Pt Interfaces. *Phys. Rev. Lett.* **115**, 267210 (2015). https://doi.org:10.1103/PhysRevLett.115.267210

4 Xiang, H. J., Kan, E. J., Wei, S.-H., Whangbo, M. H. & Gong, X. G. Predicting the spin-lattice order of frustrated systems from first principles. *Phys. Rev. B* **84**, 224429 (2011). https://doi.org:10.1103/PhysRevB.84.224429

5 Yang, J. H. *et al.* Strong Dzyaloshinskii-Moriya Interaction and Origin of Ferroelectricity in Cu_2OSeO_3. *Phys. Rev. Lett.* **109**, 107203 (2012). https://doi.org:10.1103/PhysRevLett.109.107203

6 Cui, Q., Liang, J., Shao, Z., Cui, P. & Yang, H. Strain-tunable ferromagnetism and chiral spin textures in two-dimensional Janus chromium dichalcogenides. *Phys. Rev. B* **102**, 094425 (2020). https://doi.org:10.1103/PhysRevB.102.094425

7 Kuz'min, M. D., Skokov, K. P., Diop, L. V. B., Radulov, I. A. & Gutfleisch, O. Exchange stiffness of ferromagnets. *Eur. Phys. J. Plus* **135**, 301 (2020). https://doi.org:10.1140/epjp/s13360-020-00294-y

8 Wang, Y. *et al.* Electric-field-driven non-volatile multi-state switching of individual skyrmions in a multiferroic heterostructure. *Nat. Commun.* **11**, 3577 (2020). https://doi.org:10.1038/s41467-020-17354-7

9 Zhang, H. *et al.* Room-temperature skyrmion lattice in a layered magnet $(Fe_{0.5}Co_{0.5})_5GeTe_2$. *Sci. Adv.* **8**, eabm7103 (2022). https://doi.org:doi:10.1126/sciadv.abm7103

10 Lichtenberg, T. *et al.* Anisotropic laser-pulse-induced magnetization dynamics in van der Waals magnet Fe_3GeTe_2. *2d Mater.* **10**, 015008 (2023). https://doi.org:10.1088/2053-1583/ac9dab
