# Supplementary material for: Room-temperature sub-100 nm Néel-type skyrmions in non-stoichiometric van der Waals ferromagnet Fe3-xGaTe2 with ultrafast laser writability
Source: Nat Commun. 2024 Feb 3;15:1017. doi: 10.1038/s41467-024-45310-2 (PMC10838308; doi:10.1038/s41467-024-45310-2)
Supplement: Supplementary file 3 — Description of Additional Supplementary Files [file 41467_2024_45310_MOESM3_ESM.pdf]

Description of Additional Supplementary Files

Supplementary Movie 1: Movie for detailed micromagnetic simulation on skyrmions’ laser writing process.
