# Supplementary material for: Room-temperature sub-100 nm Néel-type skyrmions in non-stoichiometric van der Waals ferromagnet Fe3-xGaTe2 with ultrafast laser writability
Source: Nat Commun. 2024 Feb 3;15:1017. doi: 10.1038/s41467-024-45310-2 (PMC10838308; doi:10.1038/s41467-024-45310-2)
Supplement: Supplementary file 5 — Reporting Summary [file 41467_2024_45310_MOESM5_ESM.pdf]

nature research

Corresponding author(s): Xuewen Fu

Lasing Reporting Summary

Nature Research wishes to improve the reproducibility of the work that we publish. This form is intended for publication with all accepted papers reporting claims of lasing and provides structure for consistency and transparency in reporting. Some list items might not apply to an individual manuscript, but all fields must be completed for clarity.

For further information on Nature Research policies, including our data availability policy, see Authors & Referees.

• Experimental design

Please check: are the following details reported in the manuscript?

1. Threshold

Plots of device output power versus pump power over a wide range of values indicating a clear threshold

- [ ] Yes
- [x] No

Our in-situ detection solely observes changes in the Lorentz phase images of the magnetic structure. The laser serves only for its thermal effect, and parameters such as the threshold are not significant.

2. Linewidth narrowing

Plots of spectral power density for the emission at pump powers below, around, and above the lasing threshold, indicating a clear linewidth narrowing at threshold

- [ ] Yes
- [x] No

Our in-situ detection solely observes changes in the Lorentz phase images of the magnetic structure. The laser serves only for its thermal effect, and parameters such as linewidth narrowing are not significant.

Resolution of the spectrometer used to make spectral measurements

- [ ] Yes
- [x] No

Our in-situ detection solely observes changes in the Lorentz phase images of the magnetic structure. The laser serves only for its thermal effect, and parameters such as linewidth narrowing are not significant.

3. Coherent emission

Measurements of the coherence and/or polarization of the emission

- [ ] Yes
- [x] No

Our in-situ detection solely observes changes in the Lorentz phase images of the magnetic structure. The laser serves only for its thermal effect, and parameters such as coherent emission are not significant.

4. Beam spatial profile

Image and/or measurement of the spatial shape and profile of the emission, showing a well-defined beam above threshold

- [ ] Yes
- [x] No

Our in-situ detection solely observes changes in the Lorentz phase images of the magnetic structure. The laser serves only for its thermal effect, and parameters such as beam spatial profile are not significant.

5. Operating conditions

Description of the laser and pumping conditions
Continuous-wave, pulsed, temperature of operation

- [x] Yes
- [ ] No

We have provided detailed descriptions of the operating conditions in both the main text and the Methods section. "The fs laser system was triggered externally with a digital delay generator which outputted single-shot fs laser pulses with 520 nm wavelength, 300 fs pulse duration and 11 mJ/cm2 fluence, where the laser spot size was adjusted to be 50 μm to ensure homogeneous illumination on the sample."

Threshold values provided as density values (e.g. W cm^{-2} or J cm^{-2}) taking into account the area of the device

- [x] Yes
- [ ] No

We have provided detailed descriptions of the operating conditions in both the main text and the Methods section. "The fs laser system was triggered externally with a digital delay generator which outputted single-shot fs laser pulses with 520 nm wavelength, 300 fs pulse duration and 11 mJ/cm2 fluence, where the laser spot size was adjusted to be 50 μm to ensure homogeneous illumination on the sample."

6. Alternative explanations

Reasoning as to why alternative explanations have been ruled out as responsible for the emission characteristics
e.g. amplified spontaneous, directional scattering; modification of fluorescence spectrum by the cavity

- [ ] Yes
- [x] No

Our in-situ detection solely observes changes in the Lorentz phase images of the magnetic structure. The laser serves only for its thermal effect, and parameters such as amplified spontaneous and directional scattering are not significant.

7. Theoretical analysis

Theoretical analysis that ensures that the experimental values measured are realistic and reasonable
e.g. laser threshold, linewidth, cavity gain-loss, efficiency

- [x] Yes
- [ ] No

The dynamic curves based on the two-temperature model can be found in Figure S22 and Supplementary Note 8.

8. Statistics

Number of devices fabricated and tested	[x] Yes<br>[ ] No	The samples subjected to a total of five Hall measurements, one LTEM, and five MFM tests are illustrated in the graphs presented in the main text Figures 1a, Figure 2a, Figure 4, Figure S13, Figure S16, etc.
Statistical analysis of the device performance and lifetime (time to failure)	[ ] Yes<br>[x] No	We characterize the fundamental properties of the material, independent of any specific devices.
